# Efficacy of Neoadjuvant Chemotherapy DOX and XELOX Regimens for Patients with Resectable Gastric or Gastroesophageal Junction Adenocarcinoma

**DOI:** 10.1155/2021/5590626

**Published:** 2021-07-22

**Authors:** Yuan Tian, Qun Zhao, Yong Li, Liqiao Fan, Zhidong Zhang, Xuefeng Zhao, Bibo Tan, Dong Wang, Peigang Yang

**Affiliations:** Department of General Surgery, The Fourth Affiliated Hospital, Hebei Medical University, No.12, Jian-Kang Road, Shijiazhuang 050019, China

## Abstract

**Purpose:**

This paper is aimed at comparing the short-term efficacy of the combination of docetaxel, oxaliplatin, and capecitabine (DOX) with the combination of oxaliplatin and capecitabine (XELOX) as neoadjuvant chemotherapy regimens for the treatment of patients with resectable gastric or gastroesophageal junction adenocarcinoma.

**Methods:**

A total of 300 patients aged 20-60 years with resectable gastric or gastroesophageal junction adenocarcinoma who were evaluated with cT3/4Nany were randomly assigned into 3 groups: DOX group (*n* = 100, treated with neoadjuvant DOX plus adjuvant XELOX), XELOX group (*n* = 100, treated with perioperative XELOX), and surgery group (*n* = 100, treated with adjuvant XELOX).

**Results:**

A total of 93, 92, and 95 patients were enrolled in the DOX, XELOX, and surgery groups, respectively. The pathological complete response (pCR) rate was 16.1% in the DOX group and 4.3% in the XELOX group (*P* = 0.008). There were 56 (61.3%) patients in the DOX group who presented with surgical complications, 22 (23.9%) patients in the XELOX group, and 37 (38.9%) patients in the surgery group. The most common grade 3-4 adverse events in these three groups were neutropenia (32.3%, 30.4%, and 21.1%), leucopenia (21.5%, 22.8%, and 15.8%), nausea (15.1%, 16.3%, and 12.6%), and fatigue (10.8%, 7.6%, and 8.4%).

**Conclusions:**

Neoadjuvant DOX is an effective and feasible regimen and might represent an option for young and middle-aged patients with locally advanced, resectable gastric or gastroesophageal junction adenocarcinoma.

## 1. Introduction

More than 50% of gastric cancer (GC) patients are neglected and diagnosed with a locally advanced stage as there is a lack of specific screening programs. Moreover, even in the cases of curative resection, the prognosis of patients with positive lymph node metastases remains poor, with five-year survival rates of 20-30% [1]. The effect of neoadjuvant chemotherapy for advanced GC has been accepted by more and more clinicians. According to NCCN guidelines [2], the FLOT regimen (docetaxel, oxaliplatin, fluorouracil, and leucovorin) was recommended. However, in the prospective clinical trials of the FLOT regimen [3–6], rare Asian patients are included, and the efficacy and tolerance of the regimen to Chinese GC patients remain unclear. Our previous clinical trial (NCT01516944) has shown the efficacy and safety of the XELOX regimen (oxaliplatin and capecitabine) and SOX regimen (tegafur, gimeracil, and oteracil potassium (S-1) plus oxaliplatin) as neoadjuvant chemotherapy [7, 8]. Docetaxel is the key treatment for metastatic GC drugs. Based on previous results [8], whether the docetaxel-based triplet chemotherapy DOX regimen can improve the pathological complete response (pCR) rate, prolong the survival, and obtain better tolerance or not needs to be confirmed. Considering potential better efficacy and more toxicity of triplet chemotherapy compared with doublet chemotherapy, middle-aged and young patients were enrolled in this study. Pathologic tumor response (pCR) has been shown as a controversial but promising survival marker in GC. Therefore, this paper mainly focuses on the short-term efficacy pCR rate.

## 2. Materials and Methods

### 2.1. Study Design and Participants

This study was an investigator-initiated multicenter, randomized, open-label, and controlled trial, and it was conducted in accordance with the principles of the Helsinki declaration. This trial was approved by the Medical Ethics Committee of the Fourth Hospital of Hebei Medical University (No. 2014020) and registered on ClinicalTrials.gov (NCT02555358, 21/09/2015). All 300 patients were centrally randomized 1 : 1 : 1 to neoadjuvant DOX plus adjuvant XELOX or perioperative XELOX or adjuvant XELOX by using an interactive web-response system (IWRS). Patients were enrolled by authorized individuals who requested randomization with an IWRS integrated into the electronic case report forms (eCRF). Assignment to trial groups was completed on the server of the independent data management providers (Bioknow, Beijing, China) via a validated assignment program, which underlies strict access control. The randomization system assigned each patient a unique identification number and sent the researchers a message containing the results of the assignment.

Patients were enrolled according to the following inclusion criteria: histologically or cytologically proved operable advanced gastric adenocarcinoma, being identified as a potentially resectable patient (cT3-4,Nany,M0) by a multidisciplinary consultation, Karnofsky Performance Scale (KPS) score > 80, Eastern Cooperative Oncology Group (ECOG) performance status of 0-1, expected survival > 6 months, age 20–60 years, voluntary participation in the study and signing the informed consent forms, and adequate major organ functions (neutrophil count ≥ 1.5 × 10^9^/L, platelet count ≥ 100 × 10^9^/L, hemoglobin ≥ 90 g/L, liver function < 1.5 times of the upper limit of normal, serum bilirubin ≤ 1.0 × UNL, serum creatinine < 1.5 × UNL, and PT-INR/PTT < 1.7 times of the upper limit of normal).

### 2.2. Procedures

Neoadjuvant DOX or XELOX was administered for 4 cycles followed by 4 cycles of postoperative XELOX for the DOX or XELOX group. Eight XELOX postoperative cycles were administered for the surgery group. DOX consisted of docetaxel 60 mg/m^2^ intravenously on day 1, oxaliplatin 130 mg/m^2^ intravenously on day 1, and capecitabine 1000 mg/m^2^ p.o. (two doses of 500 mg/m^2^ per day) on day 1 to 14, every 3-week cycle. XELOX included oxaliplatin 130 mg/m^2^ intravenously on day 1 and capecitabine 1000 mg/m^2^ given orally (500 mg/m^2^ twice a day) on day 1 to 14, every 3-week cycle. In patients with febrile neutropenia (despite the use of granulocyte colony-stimulating factor (G-CSF)), thrombocytopenia that causes bleeding, or any other hematological dose-limiting toxicities, dosing of docetaxel and oxaliplatin reduced to 75%. For grade > 2 nonhematological toxicities, the dose of all drugs was reduced to 75%; for grade 2, the dose was reduced to 50% if toxicity recurred after the first dose of reduction. Treatment continued until intolerable toxicity, disease progression or death, withdrawal of consent, or investigator's decision. Prior to surgical treatment, CT or MRI and endoscopy were performed to rule out disease progression or distant metastasis and then every 2 cycles until disease progression, recurrence, or death. The tumor volume reduction rate of 12.5% was measured by CT as an effective threshold for evaluating neoadjuvant therapy. Surgery was scheduled 4 to 6 weeks after the completion of the last cycle of neoadjuvant chemotherapy. Surgeons had to be specialized abdominal surgeons. The tumor regression grade was quantified using the NCCN Clinical Practice Guidelines in Oncology (2014.v1). TRG0 was defined as complete response without viable cancer cells, TRG1 was near complete response with single cells or rare small groups of cancer cells, TRG2 was a partial response with residual cancer cells with evident tumor regression but more than single cells or rare small groups of cancer cells, and TRG3 was poor or no response with extensive residual cancer without evident tumor regression.

Pathological staging, including depth of tumor invasion (T), lymph node involvement (N), and resection status (RX, R0, or R1), was judged by the local pathologist according to the 7^th^ edition of the TNM American Joint Committee on Cancer (AJCC) classification. Adverse events were assessed as per National Cancer Institute Common Terminology Criteria for Adverse Events (NCI-CTC, version 3.0).

### 2.3. Statistical Analysis

The sample size of this study was calculated based on the hypothesis that 5% of pCR was achieved in the XELOX group and 15% in the DOX group. A total of 300 patients were calculated to provide 80% of the power to detect this improvement in pCR (one-sided significance level of *P* < 0.05; Fisher's exact test), including 15% dropout approximately.

The analysis was conducted in the intent-to-treat (ITT) population and per-protocol (PP). Data were analyzed with SAS (version 9.3). Two-sided *P* values were calculated using Fisher's exact test, unless otherwise indicated.

## 3. Results

**Figure 1:**
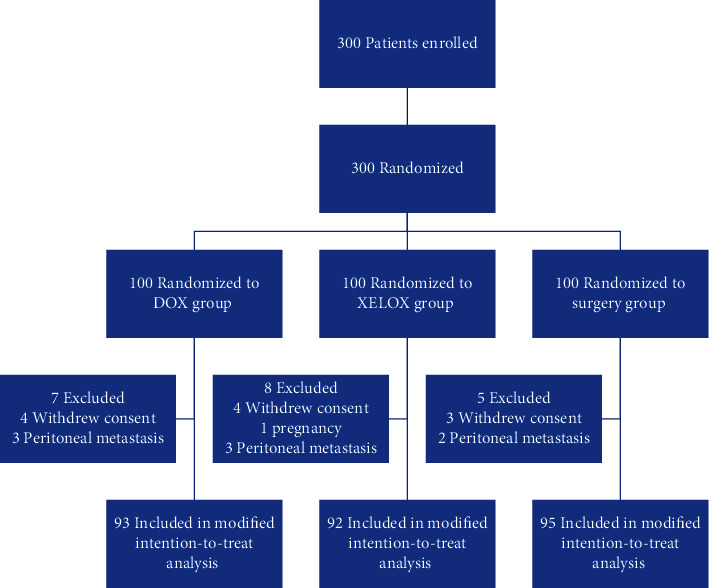
Trial profile. DOX = capecitabine, oxaliplatin, and docetaxel; XELOX = capecitabine and oxaliplatin.

Between September 2014 and June 2018, a total of 300 patients were screened (Figure 1). There were 93, 92, and 95 patients enrolled in the DOX group, the XELOX group, and the surgery group, respectively.

The baseline characteristics of the population are shown in Table 1. Patients in the DOX group included 63 males and 37 females with a median age of 52 years (range 33-60). Seventy-one males and 29 females with a median age of 54 years were included in the XELOX group. Sixty-nine males and 31 females with a median age of 52.5 years consisted the surgery group.

The average number of preoperative cycles was 3.2 in the DOX group and 3.9 in the XELOX group. There were 79 (84.9%) patients in the DOX group, 74 (80.4%) patients in the XELOX group, and 83 (87.4%) patients in the surgery group who received postoperative chemotherapy. A total of 31 (33.3%) patients in the DOX group completed the study according to the protocol (8 cycles), 37 (40.2%) patients in the XELOX group (8 cycles), and 27 (28.4%) patients in the surgery group. In addition, there were 85 (91.4%) patients who underwent surgery in the DOX group, 89 (96.7%) patients in the XELOX group, and 95 (100%) patients in the surgery group. Reasons for not proceeding to surgery were a progression of the disease, death, or metastatic disease detected after randomization, irresistibility detected during surgery, and patient request (Figure 1).

The tumor volumes before and after neoadjuvant chemotherapy in the DOX group were 52.13 ± 25.63 mm^3^ and 42.55 ± 19.31 mm^3^, respectively. The effective rate was 44.1% (14/93) of tumor volume reduction on CT. The tumor volumes before and after neoadjuvant chemotherapy in the XELOX regimen were 48.34 ± 21.56 mm^3^ and 37.32 ± 28.83 mm^3^, respectively. The effective rate was 26.1% (24/92) tumor volume reduction on CT.

All patients underwent laparoscopic exploration before the radical resection. The R0 resection rates in the DOX group, the XELOX group, and the surgery group were 97.6% (83/85), 95.5% (85/89), and 94.7% (90/95), respectively. In the DOX group, 2 patients underwent gastrojejunostomy. In the XELOX group, 2 patients underwent laparoscopy and 2 underwent palliative resection. In the surgery group, 5 patients underwent palliative resection. A significantly higher proportion of patients achieved a pathological complete regression in the DOX group than in the XELOX group (16.1% (15/93) in the DOX group *vs.* 4.3% (4/92) in the XELOX group; *P* = 0.008). The proportion of patients who achieved complete and subtotal regression was also higher with DOX (41.9% (39/93)) than with XELOX (19.6% (17/92); *P* = 0.04). The numbers of resected lymph nodes in the DOX group, the XELOX group, and the surgery group were 36.1 ± 8.4, 35.8 ± 9.2, and 39.8 ± 9.7, respectively, and this difference was not statistically significant (*t* = 1.725, *P* = 0.104). The proportion of patients with lymph node metastasis was 6.5% (218/3362) in the DOX group, 8% (252/3150) in the XELOX group, and 20.3% (767/3785) in the surgery group, and this difference was statistically significant (*t* = 108.065, *P* = 0.001).

There were 33.3% (31/93) patients in the DOX group presented with surgical complications, while 23.9% (22/92) in the XELOX group and 21.1% (20/95) in the surgery group (Table 2). Grade 3-4 nonsurgical adverse events were reported in 38 (40.9%) of 93 patients in the DOX group, 30 (32.6%) of 92 patients in the XELOX group, and 22 (23.2%) of 95 patients in the surgery group. The most frequent grade 3-4 adverse events in three groups were neutropenia (32.3% (30/93), 30.4% (28/92), and 21.1% (20/95)), leucopenia (21.5% (20/93), 22.8% (21/92), and 15.8% (15/95)), nausea (15.1% (14/93), 16.3% (15/92), and 12.6% (12/95)), and fatigue (10.8% (10/93), 7.6% (7/92), and 8.4% (8/95)) (Table 3).

## 4. Discussion

As neoadjuvant chemotherapy has been gradually accepted by clinical physicians, we found that the R0 resection is no longer difficult to achieve [9]. However, there are still quite a proportion of the patients who did not have survival benefits even with R0 resection. Compared with R0 resection, pathological remission may be a predictive parameter for survival [10–12]. Pathological regression might be a good short-term evaluation method for neoadjuvant chemotherapy for GC and a predictive parameter for survival [13].

Hence, the commonly used neoadjuvant chemotherapy regimens are difficult to meet the requirement of pathological remission. Based on the results of previous studies [8], we speculate that young Chinese patients with advanced GC with better physical conditions may improve the pathological remission rate with the treatment of a three-drug regimen of neoadjuvant chemotherapy. Therefore, the inclusion criteria of this trial were advanced GC patients under 60 years old and ECOG performance status of 0-1.

Compared with the changes in TNM staging, RECIST, and tumor volume reduction rate, pathological regression is the most accurate factor to evaluate the efficacy of neoadjuvant chemotherapy, so the primary endpoint of this trial is pathological complete regression. The proportion of patients who achieved pCR with DOX, which was 16.1% in our study, is also inconsistent with previous docetaxel-based three-drug regimen reports [14–16]; the proportion of patients who achieved pathological complete regression in these studies ranged between 14% and 20%. It is worth noting that the number of patients with complete pathological regression of DOX regimen was consistent with the response range of chemoradiation for gastroesophageal junction tumors and even exceeds that of chemoradiation [17]. For instance, the POET trial showed that 14.3% of patients with gastroesophageal junction adenocarcinoma achieved pathological complete regression with induction chemotherapy followed by chemoradiation [18]. According to the results of this study, neoadjuvant chemotherapy with either three- or two-drug regimens reduced the rate of lymph node metastasis but did not improve the rate of R0 resection.

In terms of nonsurgical adverse events, both DOX and XELOX were well tolerated and the incidences of adverse events were in line with previous reports in the perioperative setting. The main adverse reactions were leucopenia, neutropenia, and gastrointestinal symptoms [19–21].

One of the reasons for the low dropout rate in this study is that all the patients enrolled were confirmed without intraperitoneal implantation metastasis or negative for intraperitoneal abscission cytology in preoperative laparoscopic exploration. The aim is to reduce the shedding rate, which reflects the insufficiency of endoscopic and CT imaging staging and the importance of laparoscopic exploration before the treatment of GC [22]. At the same time, the DOX and XELOX groups performed laparoscopic exploration before neoadjuvant chemotherapy and before surgery again. Even so, 1 patient with positive peritoneal abscess cytology was found in each group, which indicated the importance of repeat laparoscopic exploration pre- and postneoadjuvant therapy [23].

Meanwhile, this study has some limitations. First, the selection of population was very different from real-life GC patients. The results of this study suggest that in the selection of patients for gastrectomy in metastatic esophagogastric cancer, clinical and pathological features are not enough; future prospective trials integrating tumor biology among inclusion criteria may help for defining the optimal candidates [24]. Second, pCR is an effective indicator of short-term efficacy, and whether it can prolong the overall survival of patients or not is still controversial [13, 17], which needs to be verified by further follow-up results. Third, we could not determine the optimal cycles of preoperative chemotherapy, as different cycles were not evaluated. Future trials will be needed to analyze the efficacy of different preoperative chemotherapy cycles.

In conclusion, the docetaxel-based triplet DOX significantly increased the proportion of patients achieving pathological complete regression compared with XELOX. At present, near half of the enrolled cases have benefited in terms of short-term efficacy. We will continue to recruit patients to expect more favorable results; the favorable pathological regression with DOX translates into better survival outcomes. DOX is expected to become one of the standard regimens for perioperative therapy of young and middle-aged patients with advanced GC.

## Figures and Tables

**Table 1:** General clinical characteristics of patients in the three groups.

	DOX *N* = 100	XELOX *N* = 100	Surgery *N* = 100	*χ* ^2^	*P* value
Age (year)				1.520	0.468
<45	15	17	11		
45-60	85	83	89		

Sex				0.694	0.707
M	73	69	74		
F	27	31	26		

ECOG				0.770	0.681
0	38	42	44		
1	62	58	56		

Tumor center				5.768	0.450
EGJ	33	38	28		
Gastric body	11	14	10		
Gastric antrum	52	42	53		
Other	4	6	9		

Pathological type				2.362	0.669
High and medium differentiation	56	49	51		
Poor differentiation	33	42	36		
Very low differentiation	11	9	13		

cT stage				1.510	0.470
cT3	28	31	36		
cT4	72	69	64		

cN stage				7.382	0.287
cN0	10	16	12		
cN1	35	32	41		
cN2	41	46	35		
cN3	14	6	12		

Borrmann type				9.769	0.135
I	3	0	0		
II	35	39	31		
III	55	56	66		
IV	7	5	3		

Very low differentiation: signet ring cell carcinoma, mucous adenocarcinoma, and anaplastic carcinoma.

**Table 2:** Serious adverse events with perioperative morbidity.

	DOX (*n* = 93)	XELOX (*n* = 92)	Surgery (*n* = 95)	*χ* ^2^	*P* value
Patients with at least one serious adverse event involving a perioperative morbidity	31	22	20	4.008	0.135
*Surgical complication*					
Pneumonia	8	7	7	0.11	0.946
Pleural complication	15	5	11	5.41	0.067
Chyle leakage	1	0	0	2.018	0.365
Seroperitoneum	1	1	1	0.001	1.000
Anastomotic fistula	1	1	0	1.035	0.596
Intestinal occlusion	0	0	1	1.954	0.376

**Table 3:** Adverse events in 3 groups.

	DOX 93			XELOX 92			Surgery 95		
	Grades 1-2	Grade 3	Grade 4	Grades 1-2	Grade 3	Grade 4	Grades 1-2	Grade 3	Grade 4
*Gastrointestinal disorders*									
Nausea	41	14	0	36	15	0	38	12	0
Vomiting	20	4	0	10	2	0	12	0	0
Diarrhea	34	6	0	26	2	0	16	6	0
Constipation	8	0	0	8	0	0	4	0	0
Decreased appetite	46	8	0	38	8	0	40	6	0
Dysphagia									
*Blood and lymphatic system disorders*									
Anemia	44	6	2	44	2	0	38	4	0
Leucopenia	54	16	4	50	17	4	46	15	0
Neutropenia	59	24	6	48	26	2	40	18	2
Thrombocytopenia	22	4	2	16	0	0	18	0	0
Febrile neutropenia	NA	2	0	NA	0	0	NA	0	0
*General and other disorders*									
Neurotoxic effects	14	0	0	12	0	0	8	0	0
Fatigue	46	10	0	38	7	0	34	8	0
Alopecia	58	NA	NA	0	NA	NA	0	NA	NA
Weight decreased	34	4	0	38	0	0	28	0	0
Skin effects	8	0	0	4	0	0	2	0	0
*Laboratory*									
ALT elevation	14	2	0	10	0	0	8	0	0
GOT elevation	10	2	0	12	0	0	10	0	0

## Data Availability

The [participant data with identifiers] data used to support the findings of this study were supplied by [Qun Zhao] under license and so cannot be made freely available. Requests for access to these data should be made to [Qun Zhao, zhaoqun@hebmu.edu.cn].
